# Analysing detection of chronic diseases with prolonged sub-clinical periods: modelling and application to hypertension in the U.S.

**DOI:** 10.1186/s12874-019-0845-2

**Published:** 2019-11-27

**Authors:** Ralph Brinks, Sophie Kaufmann, Annika Hoyer, Edward W Gregg, Jürgen Saal

**Affiliations:** 1 University Hospital, Department and Hiller research unit for Rheumatology, Moorenstr. 5, Düsseldorf, 40225 Germany; 2 German Diabetes Center, Institute for Biometry and Epidemiology, Auf’m Hennekamp 65, Düesseldorf, 40225 Germany; 3 Heinrich-Heine-University Düsseldorf, Mathematical Institute, Düsseldorf, Germany; 4 Centers for Disease Control and Prevention, Division of Diabetes Translation, Atlanta, Georgia, United States of America; 5 Imperial College London, School of Public Health, London, United Kingdom

**Keywords:** Compartment model, Incidence, Prevalence, NHANES

## Abstract

**Background:**

We recently introduced a system of partial differential equations (PDEs) to model the prevalence of chronic diseases with a possibly prolonged state of asymptomatic, undiagnosed disease preceding a diagnosis. Common examples for such diseases include coronary heart disease, type 2 diabetes or cancer. Widespread application of the new method depends upon mathematical treatment of the system of PDEs.

**Methods:**

In this article, we study the existence and the uniqueness of the solution of the system of PDEs. To demonstrate the usefulness and importance of the system, we model the age-specific prevalence of hypertension in the US 1999–2010.

**Results:**

The examinations of mathematical properties provide a way to solve the systems of PDEs by the method of characteristics. In the application to hypertension, we obtain a good agreement between modeled and surveyed age-specific prevalences.

**Conclusions:**

The described system of PDEs provides a practical way to examine the epidemiology of chronic diseases with a state of undiagnosed disease preceding a diagnosis.

## Background

Chronic non-communicable diseases (NCDs) have emerged as a major global burden, accounting for 40 million of the 56 million global deaths in 2016. About 18 million of those deaths were due to cardiovascular disease [1]. Although hypertension is an NCD by itself, it is also an important risk factor for cardiovascular disease, stroke and other chronic diseases like, e.g., kidney disease [2]. As hypertension is without symptoms at an early stage of the disease, an enormous number of people suffer from undiagnosed hypertension, delaying effective preventive treatment. For example, in a nationally representative survey in China Gao *et al* reported that more than 70 percent of men and women aged 20-44 years with hypertension did not have a diagnosis [3]. With a view to hypertension as a risk factor for chronic diseases, the World Health Organization has identified “detection, treatment and control of hypertension” as one of the objectives in the *Global Action Plan For the Prevention and Control of NCDs* [4].

Recently, we developed a four-state model to systematically examine how incidence and prevalence of undiagnosed chronic diseases and possible subsequent diagnosis are related [5]. The four-state model is an extended illness-death model, which additionally comprises a state of being undiagnosed before possibly transiting to the diagnosed state. The four-state model is related to a two-dimensional system of partial differential equations (PDEs). However, no rigorous analysis of the mathematical properties, e.g., classification of the type, existence and uniqueness of the solution of the system of PDEs to facilitate application and use has been published.

In this paper, we prove the existence and uniqueness of the solution of the two-dimensional system of PDEs and then apply the system of PDEs to model the age-specific prevalence of undiagnosed and diagnosed hypertension in the US.

## Methods

After a short derivation of the system of PDEs based on the four-state model, we use the method of characteristics to prove existence and uniqueness of the solution of the PDE. The method of characteristics is a classical tool in order to prove well-posedness of PDEs. It also opens a way to calculate this unique solution. Readers who are not familiar with PDEs may find introductory texts by Zachmanoglou & Thoe [6] and DuChateau & Zachmann [7].

We demonstrate usefulness of the four-state model in modelling the prevalence of undiagnosed (*p*_1_) and diagnosed (*p*_2_) hypertension for different age-groups in the period from 1999 to 2010. With reasonable assumptions about the incidence of hypertension and mortality data from the US, we show that the four-state model can achieve a good agreement with the observed prevalence data about hypertension from the nationally representative *National Health and Nutrition Examination Survey* (NHANES) in the US. The assumptions about the incidence and mortality rates are detailed in the next section. The reason why we have to make (reasonable) assumptions – instead of using published data – is that the required data are not available. Especially, the mortality of people with undiagnosed hypertension is difficult to survey.

In NHANES, hypertension was defined as systolic blood pressure ≥ 140 mm Hg or diastolic blood pressure ≥ 90 mm Hg, or being on antihypertensive medication. Age-specific prevalence of hypertension (*p*_1_+*p*_2_) has been reported for the years 1999 to 2010. Awareness of hypertension has also been surveyed. Awareness was defined as the fraction of the population who has been informed of a hypertension diagnosis. Thus, awareness corresponds to the fraction $\tfrac {p_{2}}{p_{1}+p_{2}}$. This information allows calculation of age-specific prevalence of undiagnosed and diagnosed hypertension. It is not our aim to make the best possible fit between the modelled and the observed prevalences, but rather to show that a reasonable fit is easily possible. As we do not intend the best fit, which indeed could be the subject of a paper on its own, readers should not be tempted to make inferences about the underlying epidemiological rates.

## Results

### The chronic disease model with four states

**Fig. 1:**
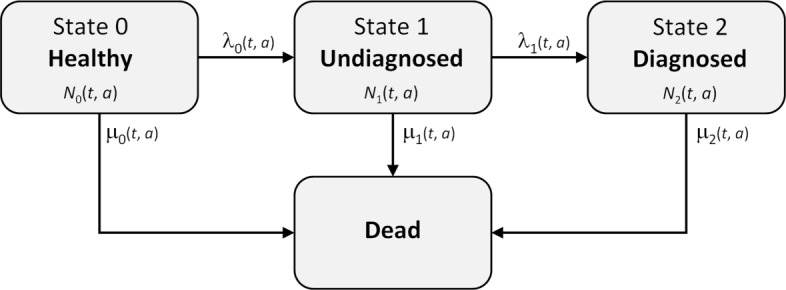
Illness-death model with a state of undiagnosed disease. The transition rates between the four states (*λ*_*i*_,*μ*_*j*_,*i*=0,1,*j*=0,1,2) depend on calendar time *t* and age *a*

To analyse a population with respect to the chronic disease, we consider the compartment model from our previous work [5] as shown in Fig. 1. The model is the well-known illness-death model [8] with an additional state that comprises people with undiagnosed disease. The numbers *N*_*j*_, *j*=0,1,2, as well as the transition rates shown in Fig. 1 depend on the calendar time $t \in \mathbb {R}$ and on the age *a*, *a*∈[0,*∞*). *N*_*j*_(*t*,*a*) denotes the number of people in state *j*, *j*=0,1,2, aged *a* at time *t*.

With the assumption that there is no migration, we have shown in [5] that the numbers *N*_*j*_ are solutions of the following system of partial differential equations (PDEs):

1 $$\begin{array}{@{}rcl@{}} {}(\partial_{t} + \partial_{a}) \, N_{0}(t,a) &=& -(\mu_{0}(t,a) + \lambda_{0}(t,a)) \, N_{0}(t,a),  \end{array} $$

2 $$\begin{array}{@{}rcl@{}} {}(\partial_{t} + \partial_{a}) \, N_{1}(t,a) &=& -(\mu_{1}(t,a) + \lambda_{1}(t,a)) \, N_{1}(t,a)\\ &&+ \lambda_{0}(t,a) \, N_{0}(t,a),  \end{array} $$

3 $$\begin{array}{@{}rcl@{}} {}(\partial_{t} + \partial_{a}) \, N_{2}(t,a) &=& -\mu_{2}(t,a) \, N_{2}(t,a)\\ &&+ \lambda_{1}(t,a) \, N_{1}(t,a).  \end{array} $$

For brevity, we have written *∂*_*t*_ for $\tfrac {\partial }{\partial t}$ and *∂*_*a*_ for $\tfrac {\partial }{\partial a}$. In addition, we set *N*(*t*,*a*):=*N*_0_(*t*,*a*)+*N*_1_(*t*,*a*)+*N*_2_(*t*,*a*) for the overall number of people aged *a* at time *t*.

### System of PDEs for the age-specific prevalence

In chronic disease epidemiology, it is common to consider the fractions of people who are in the disease states instead of their absolute numbers *N*_*j*_. For this, set $p_{j}(t,a):=\tfrac {N_{j}(t,a)}{N(t,a)}$ for *j*=0,1,2. By using

$$(\partial_{t} + \partial_{a}) p_{j}(t,a) = \frac{(\partial_{t} + \partial_{a}) N_{j}(t,a)}{N(t,a)} + \mu(t,a) p_{j}(t,a) $$

and defining the overall mortality *μ*=*μ*_0_*p*_0_+*μ*_1_*p*_1_+*μ*_2_*p*_2_=*μ*_0_(1−*p*_1_−*p*_2_)+*μ*_1_*p*_1_+*μ*_2_*p*_2_, we can deduce the following PDEs from Eqs. (2) and (3).

4 $$\begin{array}{@{}rcl@{}} {}(\partial_{t} + \partial_{a}) p_{1} &=& - (\lambda_{0} + \lambda_{1} + \mu_{1} - \mu) p_{1} - \lambda_{0} p_{2} + \lambda_{0}, \end{array} $$

5 $$\begin{array}{@{}rcl@{}} {}(\partial_{t} + \partial_{a}) p_{2} &=& \lambda_{1} p_{1} - (\mu_{2} - \mu) p_{2}.  \end{array} $$

Instead of the three Eqs. (1) – (3), only two equations are necessary to describe the model in Fig. 1. The fraction *p*_0_ can be obtained from the equation *p*_0_+*p*_1_+*p*_2_=1. Eqs. (4) and (5) define a two-dimensional system of linear PDEs.

#### **Remarks 1**

We notice that Eqs. (4) and (5) actually represent a nonlinear system by the fact that *μ*=*μ*_0_*p*_0_+*μ*_1_*p*_1_+*μ*_2_*p*_2_. However, in practice the mortality rate *μ* of a population can be deduced from empirical data. Thus, we can assume (4) and (5) to be linear.

With the definitions

$$\begin{array}{*{20}l} \boldsymbol{p} &:= \left(\begin{array}{c} p_{1}\\ p_{2} \end{array}\right), \; I := \left(\begin{array}{cc} 1 & 0 \\ 0 & 1 \end{array}\right), \, \boldsymbol{b} := \left(\begin{array}{c} b_{1} \\ b_{2} \end{array}\right)\\ &= \left(\begin{array}{c} -(\lambda_{0} + \lambda_{1} + \mu_{1} - \mu) p_{1} - \lambda_{0} p_{2} - \lambda_{0} \\ \lambda_{1} p_{1} - (\mu_{2} - \mu) p_{2} \end{array}\right), \end{array} $$

Eqs. (4) and (5) read as

6 $$ I \; \partial_{t} \boldsymbol{p}(t,a) + I \; \partial_{a} \boldsymbol{p}(t, a) = \boldsymbol{b}(t, a, \boldsymbol{p}).  $$

Since *I* is the identity matrix, system (6) is hyperbolic [7].

Now we mimic the *method of characteristics* for systems of PDEs [6*,*^9^]. We consider the initial curve $\mathbb {R} \ni t\mapsto \gamma (t) := (\gamma ^{(1)}(t), \gamma ^{(2)}(t)) := (t,0) \in \mathbb {R}^{2}$ and assume for the time being that *λ*_*j*_, *μ*_*j*_, and *μ* are sufficiently smooth. Then, we have

7 $$ (\gamma^{(1)})'(t)-(\gamma^{(2)})'(t)=1\neq 0\quad (t\in\mathbb{R})  $$

which shows that *γ* is not characteristic.

The *characteristic curves* *y*=(*y*^(1)^,*y*^(2)^) in the *t*-*a*-plane along *γ* are determined by [6*,*^9^]

$$\begin{array}{*{20}l} y^{(1)}(s)&=1,\quad y^{(1)}(0)=\gamma^{(1)}(t_{0})=t_{0},\\ y^{(2)}(s)&=1,\quad y^{(2)}(0)=\gamma^{(2)}(t_{0})=0. \end{array} $$

This system is solved by $y_{t_{0}}(s)=(t_{0}+s,s)$. Setting $\psi (t_{0},s):=y_{t_{0}}(s)\phantom {\dot {i}\!}$, we obtain for its inverse *ψ*^−1^(*t*,*a*)=(*t*−*a*,*a*). The solution of Eqs. (4) and (5) hence is given as

8 $$ \boldsymbol{p}(t,a)=V(\psi^{-1}(t,a)),  $$

where *V*(*t*_0_,·) represents the solution of the ordinary differential equation (ODE)

9 $$ {}\frac{\mathrm{d} V(t_{0},s)}{\mathrm{d} s} = \boldsymbol{b}(y_{t_{0}}(s),V(t_{0},s)), \quad V(t_{0},0)=\boldsymbol{p}_{0}(t_{0})  $$

where $\boldsymbol {p}_{0}(t_{0})=\boldsymbol {p}(t_{0},0) \in \mathbb {R}^{2}$ is a given initial value.

**Fig. 2:**
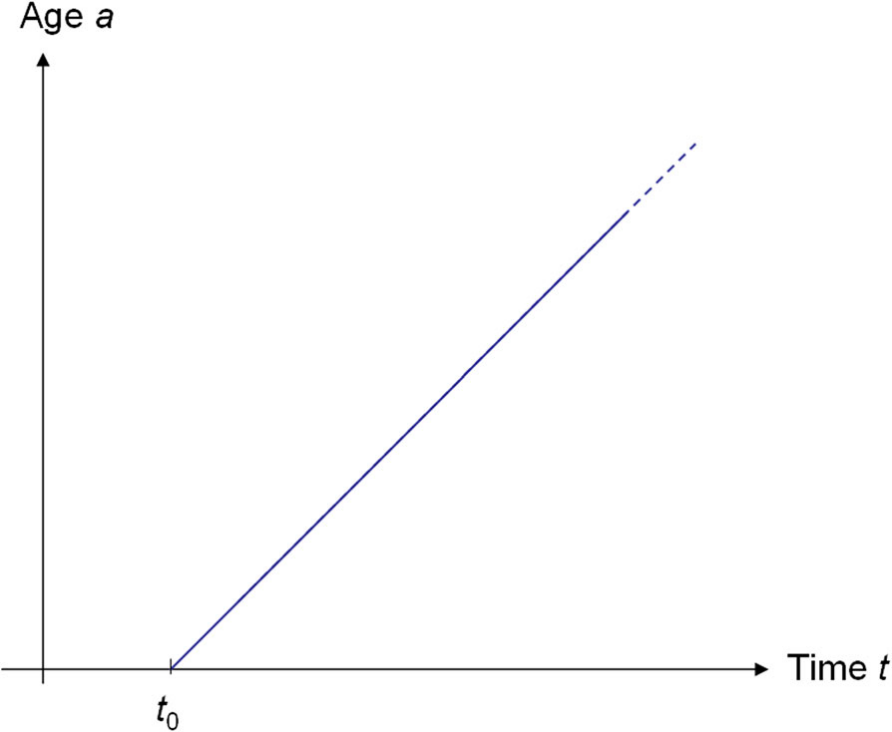
Lexis diagram with a life line. The life line (oblique) represents a birth cohort born at *t*=*t*_0_ in the Lexis diagram (*t*-*a*-plane). The life lines are the characteristic curves of system (6)

Before we utilize the outcome on existence and uniqueness of the solution of the PDE system (6) obtained so far, we describe the geometrical meaning of (9). Note that the calculation above motivates to identify *a*=*s*. Then, for $t_{0} \in \mathbb {R}$ the line segment given by $y_{t_{0}}(a) = (t_{0} + a, a), ~a\in [0, \infty)$, is a characteristic curve for (6). One of these line segments in the *t*-*a*-plane is shown in Fig. 2. The line segment starts at (*t*,*a*)=(*t*_0_,0) and has slope 1. The line segment can be seen as the trajectory of a group of persons born at the same point in time *t*_0_ (birth cohort) which gradually grows older. In demography and less frequently also in epidemiology, such a representation of the *t*-*a*-plane is called a *Lexis diagram* [10]. Line segments with slope 1 starting on the abscissa like the one depicted in Fig. 2 are called *life lines* [11]. Now, we notice that the system of ODEs (9) can be written in terms of ***p*** as

10 $$ {}\frac{\mathrm{d} \boldsymbol{p}(y_{t_{0}}(a))}{\mathrm{d} a} = \boldsymbol{b}(y_{t_{0}}(a),\boldsymbol{p}(y_{t_{0}}(a))), \quad \boldsymbol{p}(y_{t_{0}}(0))=\boldsymbol{p}_{0}(t_{0}).  $$

With this terminology, we see that system (10) describes the change of the prevalence ***p*** along the life lines in the Lexis diagram.

Next, we state the existence and uniqueness of the solution of the PDE system (6) as a theorem. We consider two kinds of initial curves depending on the domain *S* where the right hand side of Eq (6) is defined.

1. The domain *S* is the upper half-plane, i.e., $S = \mathbb {R} \times [0, \infty)$. Then, the initial condition is given on the real line defined by *a*=0, i.e., for all $(t, a) \in \delta S : = \mathbb {R} \times \{0\}$. Here the initial condition reads
  11 $$ \boldsymbol{p}(t,0)=\boldsymbol{p}_{0}(t)\quad (t\in\mathbb{R}).  $$
2. The domain *S* is the first quadrant, i.e., *S*=[0,*∞*)^2^. Then, the initial condition is given on the union of two orthogonal half-lines, i.e., for all $(t, a) \in \delta S : = \bigl ([0, \infty) \times \{0\} \bigr) \cup \bigl (\{0\} \times [0, \infty) \bigr)$. In this case the initial conditions are given as
  12 $$ {}\boldsymbol{p}(t, 0) = \boldsymbol{p}_{0}(t)\quad (t>0)\quad\text{and}\quad \boldsymbol{p}(0, a) = \boldsymbol{p}_{1}(a)\quad (a>0).  $$

#### **Theorem 1**

Let the rates $\lambda _{i}, \mu _{j}, \mu : \bar S \rightarrow [0, \infty)$ be continuously differentiable for *i*=0,1 and *j*=1,2, where $\bar S$ denotes the closure of *S*. Furthermore, let ***p***_0_:[0,*∞*)→[0,1]^2^ be continuously differentiable. In case that *S* is the first quadrant also assume that ***p***_1_:[0,*∞*)→[0,1]^2^ is continuously differentiable and that the compatibility conditions ***p***_0_(0)=***p***_1_(0) and ***p***0′(0)=***p***1′(0) are satisfied. Then the system (6) with initial condition (11), if *S* is the upper half-plane, or with initial condition (12), if *S* is the first quadrant, has a unique continuously differentiable solution $\boldsymbol {p}:\bar S\to \mathbb {R}^{2}$.

#### *Proof*

In the same way as in (7) it can be seen that also the initial hyper plane *δS* is not characteristic in case that *S* is the first quadrant. Due to the given assumptions on the data, by the Picard-Lindelöf Theorem the system (9) is uniquely solvable. Thus, the solution of (6) can be constructed as demonstated above, that is, it is given by (8) and has the claimed regularity. □

The equivalence between the systems (6) and (10) point out a possible way to calculate the unique solution of system (6) with initial condition (11) or (12), respectively. For this purpose, classical numerical methods for systems of ODEs like, e.g., the Runge-Kutta method, can be used [12]. This will be demonstrated in the next section.

### Undiagnosed and diagnosed hypertension

**Fig. 3:**
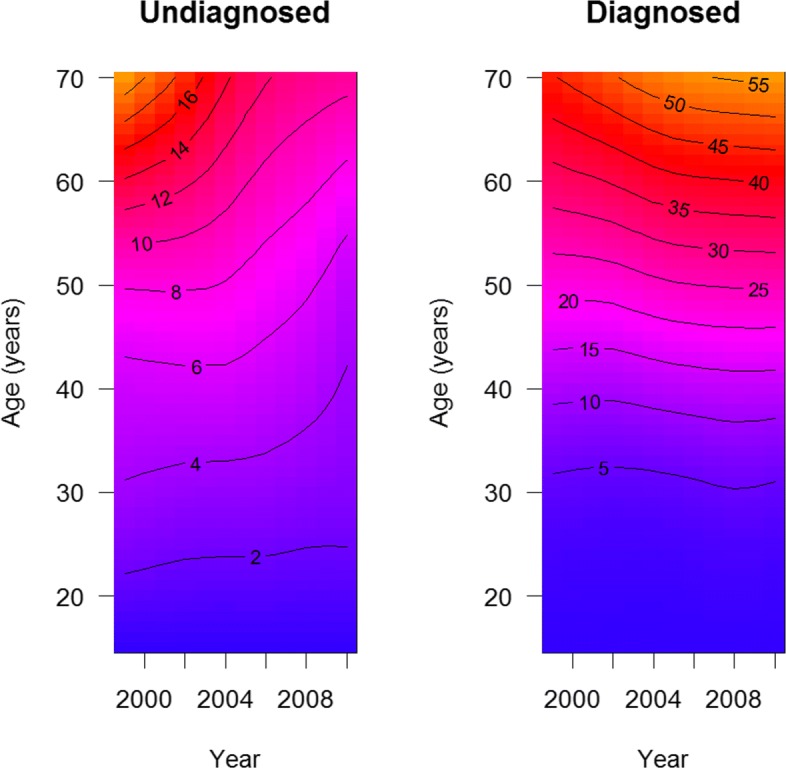
Prevalence of undiagnosed (left) and diagnosed hypertension (right). The prevalence of undiagnosed and diagnosed hypertension (in percent) is shown as color and contour lines for different ages (*a*) and times (*t*)

Figure 3 shows the prevalence of undiagnosed (left) and diagnosed hypertension (right) in the age range 18-70 years during the years 1999-2010 as surveyed in NHANES [13].

Similar to the Lexis diagram, the abscissa and ordinate represent the calendar time (*t*) and the age (*a*), respectively. The colour and the contour lines indicate the prevalence (in percent). For instance, the prevalence of undiagnosed hypertension for 60 year old people in the year 2000 was about 14%. In 2006, the prevalence of undiagnosed hypertension decreased to about 10% for people aged 60. During the same period, the prevalence of diagnosed hypertension for 60 year old people has increased from slightly less then 35% to about 40%.

Now, we calculate the unique solution of the system (6) for (*t*,*a*)∈[1999,2010]×[0,70]. As initial condition, we chose ***p***(*t*,0)=(0,0) for all *t*≥1999 and ***p***(1999,*a*)=***p***_0_(*a*) for all *a*≥0. Here, ***p***_0_(*a*) is the age-specific prevalence as surveyed in 1999 [13]. We have an initial condition given on two half-lines. The mortality rate *μ* of the US general population for the period 1999–2010 has been taken from the *Human Mortality Database* [14]. For the mortality rates in the states *Undiagnosed Hypertension* (*μ*_1_) and *Diagnosed Hypertension* (*μ*_2_), we assume *μ*_*j*_=*R*_*j*_ *μ*, *j*=1,2, where *R*_1_=1.1 and *R*_2_=1.2, respectively. Currently, there are no data about mortality of people with undiagnosed and diagnosed hypertension compared to the general population. Based on NHANES data, values between 1.09-1.49 have been reported for untreated hypertension compared to controlled hypertension [15]. Thus, the magnitude of our choice seems reasonable. However, the exposition states in [15] are differently defined from our model (see Fig. 1). Moreover, we believe that these values are slightly overestimated because the study design of [15] cannot not take into account possible changes from *Untreated Hypertension* to *Controlled Hypertension* after baseline. Hence, people untreated at baseline may later be treated and may thus have a reduced mortality with this treatment. The incidence rates *λ*_0_ and *λ*_1_ have been determined by decomposing these rates into a time-dependent factor $\lambda ^{(T)}_{j}$ and an age-dependent factor $\lambda ^{(A)}_{j}$ [16]:

$$\lambda_{j}(t, a) = \lambda^{(T)}_{j}(t) \times \lambda^{(A)}_{j}(a), ~j=0, 1.$$

Although there are systematic ways to estimate the rates *λ*_*j*_, *j*=0,1, as described in [5], we only made coarse guesses for $\lambda ^{(A)}_{j}$ and $\lambda ^{(T)}_{j}, ~j=0,1,$ such that the modelled prevalence approximates the surveyed prevalence (see Fig. 3). The source code for running the calculations to be run with the freely available statistical software R (The R Foundation for Statistical Computing) is given as Additional file 1.

**Fig. 4:**
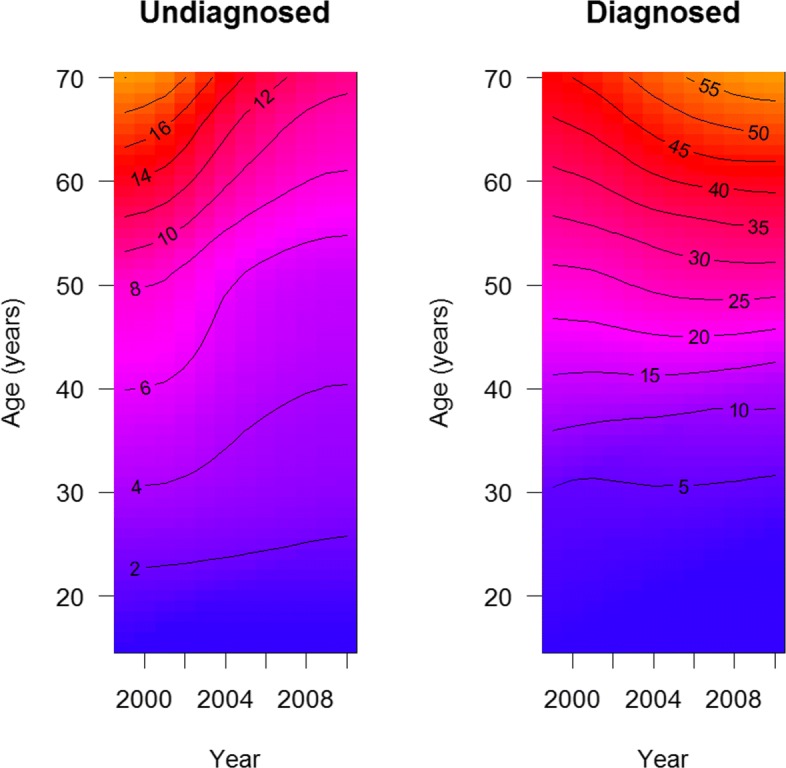
Modelled prevalence of undiagnosed (left) and diagnosed hypertension (right). The modelled prevalence of undiagnosed and diagnosed hypertension is shown as the corresponding surveyed prevalence in Fig. 3

Figure 4 shows the modelled prevalence that has been obtained by solving the initial value problem described above. After transforming the two dimensional PDE (6) with initial condition into the corresponding initial value problem of the ODE (10), the classical Runge-Kutta method of fourth order has been applied to calculate ***p***(*t*,*a*) for (*t*,*a*)∈[1999,2010]×[0,70].

**Fig. 5:**
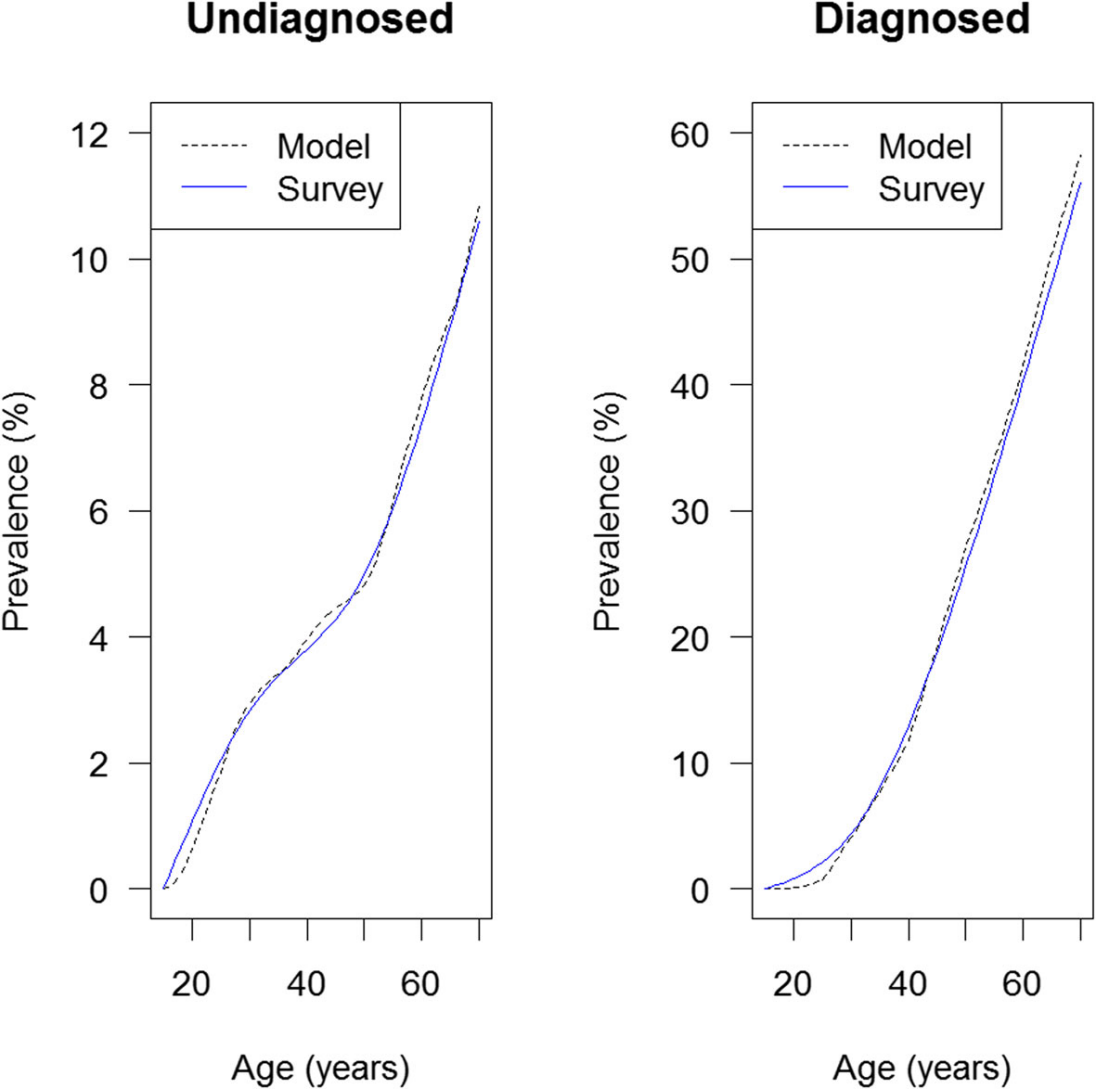
Surveyed and modelled age-specific prevalence of undiagnosed (left) and diagnosed hypertension (right) at year *t*=2010. The surveyed and modelled prevalence are shown as solid blue and dashed black lines, respectively

Overall we see a good agreement between the surveyed and the modelled prevalence. For a direct comparison we plot the surveyed and the modelled age-specific prevalence for the year *t*=2010 in Fig. 5.

## Discussion

In this article, we have proven the existence and uniqueness of the solution of a recently published system of PDEs that describes the prevalence of undiagnosed and diagnosed chronic diseases. The proof uses the method of characteristics to transform the initial value problem of the PDE into an associated initial value problem of an ODE. Apart from the theoretical considerations, the method of characteristics provides a practical way to calculate the unique solution of the initial value problem. We have demonstrated this method in an example about hypertension in the US. The solution of the initial value problem agrees well with the observed prevalence data of hypertension obtained from a representative sample of the US population. Undiagnosed hypertension is a problem in the US and many other populations, because it is a risk factor for several severe health conditions such as stroke, cardiovascular disease and kidney disease.

In epidemiological applications of the proposed framework, input data usually are subject to statistical uncertainties, e.g., due to possible sampling errors. In order to solve the system of PDE in the presence of uncertainty, we suggest to use a multidimensional probabilistic approach, which randomly samples from the probability distributions of the input parameters, solves the PDEs (4) and (5) based on these samples, and then assesses the distribution of the results. The underlying ideas are detailed by Oakley and O’Hagan [17] and have been successfully applied in a public health setting [18].

Our work has several advantages and disadvantages. On the one hand, the disease model is relatively generic and can be applied to any chronic disease with a considerable state of undiagnosed disease. No assumptions about the form of the involved transition rates in the model have been made. In this way, the model is non-parametric.

In its current form, the model assumes that there is no migration from or into the considered population, which might be seen as a drawback. However, additional rates representing immigration or emigration can be added to Eqs. (4) and (5) following the corresponding considerations as in the normal illness-death model (without the undiagnosed state) [19]. Another drawback is that some of the epidemiological figures of the disease model are difficult to estimate in practice. While the age-specific prevalence of undiagnosed and diagnosed hypertension can easily be surveyed by cross-sectional studies, estimation of the mortality rates for undiagnosed and diagnosed hypertension is difficult. The study design of NHANES includes a linkage with the US mortality register. However, changes of the hypertension status between the NHANES examination and death (from no hypertension to undiagnosed hypertension, from undiagnosed to diagnosed hypertension) cannot be taken into account. This possibly leads to a misclassification error where death cases are attributed to the wrong disease state. A theoretical alternative might be a cohort study to assess the mortality of undiagnosed hypertension (*μ*_1_). However, keeping the information of survey-detected hypertension secret from a study subject without previous diagnosis of hypertension would be unethical. For our purpose of giving a demonstration about a possible application, we have made reasonable assumptions about the mortality rates *μ*_1_ and *μ*_2_ from the hypertension states.

The aim of our application to hypertension was to demonstrate usefulness of the disease model and the associated PDEs. Obtaining the highest degree of consistency between our modelled prevalence and the surveyed prevalence was not intended. Hence, the results should be used carefully for drawing conclusions about public health relevant questions.

The four-state model and the associated PDEs have a variety of possible applications. For example, the model may help to understand which age groups should be taken special care of with respect to detection. When the model is stratified by subgroups of the considered population, e.g., by ethnicity, education, socio-economic position etc., decision makers may obtain information about especially vulnerable parts of the population. This may form the basis for potential screening and intervention programmes. The impact of a potential screening programme for hypertension and other chronic diseases with prolonged states of undiagnosed disease such as coronary heart disease or cancer may be analyzed in advance.

Another straightforward application of the four-state model and the associated PDE would be a prediction of future prevalence of undiagnosed and diagnosed hypertension using what-if scenarios. For example, it is possible to predict the consequences of different future time trends of the incidence of hypertension.

Finally, the model may help to analyse temporal trends of transition rates *λ*_0_ and *λ*_1_ between the states, which has been demonstrated in [5]. This question is important for assessing the quality of case-finding in the epidemiology of chronic diseases. Usually, prevalence based measures have been used for assessing case-finding [20]. However, we have shown recently that measures based on transition rates are more reliable [21].

## Conclusions

In this article we have shown the existence and uniqueness of the solution of a system of partial differential equations that describes an extended illness-death model. Based on the usual illness-death model, a state of undiagnosed disease has been added, which can be used to model chronic diseases with a (possibly) prolonged state of undiagnosis preceding a diagnosis. As an example, we applied the model to hypertension in the US.

## Supplementary information

**Additional file 1** Script for the statistical software r. The text-file contains the commands and analysis for the simulation.

## Data Availability

The data used in this article were published in [13]. No further collection of individual persons’ data has been accomplished. All results can be reproduced from the source files provided as Additional file 1. The source files can be run with the freely available statistical software R (The R Foundation for Statistical Computing).

## Abbreviations

NCD: non-communicable disease
NHANES: National Health and Nutrition Examination Survey
ODE: ordinary differential equation
PDE: partial differential equation
